# First large-scale study reveals important losses of managed honey bee and stingless bee colonies in Latin America

**DOI:** 10.1038/s41598-024-59513-6

**Published:** 2024-05-02

**Authors:** Fabrice Requier, Malena Sibaja Leyton, Carolina L. Morales, Lucas A. Garibaldi, Agostina Giacobino, Martin Pablo Porrini, Juan Manuel Rosso-Londoño, Rodrigo A. Velarde, Andrea Aignasse, Patricia Aldea-Sánchez, Mariana Laura Allasino, Daniela Arredondo, Carina Audisio, Natalia Bulacio Cagnolo, Marina Basualdo, Belén Branchiccela, Rafael A. Calderón, Loreley Castelli, Dayson Castilhos, Francisca Contreras Escareño, Adriana Correa-Benítez, Fabiana Oliveira da Silva, Diego Silva Garnica, Grecia de Groot, Andres Delgado-Cañedo, Hermógenes Fernández-Marín, Breno M. Freitas, Alberto Galindo-Cardona, Nancy Garcia, Paula M. Garrido, Tugrul Giray, Lionel Segui Gonçalves, Lucas Landi, Daniel Malusá Gonçalves, Silvia Inés Martinez, Pablo Joaquín Moja, Ana Molineri, Pablo Fernando Müller, Enrique Nogueira, Adriana Pacini, María Alejandra Palacio, Guiomar Nates Parra, Alejandro Parra-H, Kátia Peres Gramacho, Eleazar Pérez Castro, Carmen Sílvia Soares Pires, Francisco J. Reynaldi, Anais Rodríguez Luis, Carmen Rossini, Milton Sánchez Armijos, Estela Santos, Alejandra Scannapieco, Yamandú Mendoza Spina, José María Tapia González, Andrés Marcelo Vargas Fernández, Blandina Felipe Viana, Lorena Vieli, Carlos Ariel Yadró García, Karina Antúnez

**Affiliations:** 1 Université Paris-Saclay, CNRS, IRD, UMR Évolution, Génomes, Comportement et Écologie, 91198 Gif-sur-Yvette, France; 2 Sociedad Latinoamericana de Investigación en Abejas (SOLATINA), Montevideo, Uruguay; 3 Grupo Ecología de la Polinización, INIBIOMA (CONICET-Universidad Nacional del Comahue), Quintral 1250, Bariloche, Río Negro Argentina; 4 Universidad Nacional de Río Negro, Instituto de Investigaciones en Recursos Naturales, Agroecología y Desarrollo Rural, Bariloche, Río Negro Argentina; 5 Consejo Nacional de Investigaciones Científicas y Técnicas, Instituto de Investigaciones en Recursos Naturales, Agroecología y Desarrollo Rural, Bariloche, Río Negro Argentina; 6 Instituto de Investigación de La Cadena Láctea (INTA-CONICET), Estación Experimental Agropecuaria- Rafaela, Ruta 34 Km 227, 2300 Rafaela, Santa Fe Argentina; 7 Centro de Investigaciones en Abejas Sociales (CIAS)-Instituto de Investigación en Producción Sanidad y Ambiente (IIPROSAM CONICET-UNMdP), Facultad de Ciencias Exactas y Naturales, Centro Científico Tecnológico Mar del Plata-CONICET, Centro de Asociación Simple CIC PBA, Estación Costera J.J. Nágera, Ruta Provincial 11 Km 5395 Playa Chapadmalal (7603) Mar del Plata, Buenos Aires, Argentina; 8 Universidad Distrital Francisco José de Caldas, Facultad de Medio Ambiente y Recursos Naturales and Colectivo Abejas Vivas, Bogotá, Colombia; 9 Ministerio de la producción y ambiente Formosa (MPA), Facultad de Recursos Naturales, Universidad de Formosa (UNAF), Av Luís Gutnisky 3200, Formosa, Argentina; 10 Universidad SEK, Facultad de Ciencias de la Salud, Instituto de Investigación Interdisciplinar en Ciencias Biomédicas SEK, Santiago, Chile; 11 Área de Investigación y Desarrollo Tecnológico para la Agricultura Familiar Región Cuyo, INTA, San Juan entre Sarmiento y José Pedro Cortinez Oeste, San Martín, 5439 San Juan, Argentina; 12 Lab. de Microbiología y Salud de las Abejas, Departamento de Microbiología, Instituto de Investigaciones Biológicas Clemente Estable, Montevideo, Uruguay; 13 Instituto de Investigaciones para la Industria Química (INIQUI-CONICET), Universidad Nacional de Salta, Av. Bolivia 5150, Salta, Argentina; 14 Facultad de Ciencias Veterinarias-PROANVET Universidad Nacional del Centro de la Provincia de Buenos Aires UNCPBA, Tandil, Buenos Aires, Argentina; 15 Sección Apicultura, Instituto Nacional de Investigación Agropecuaria, Ruta 50, km 11, Colonia, Uruguay; 16 Programa Integrado de Patología Apícola, Centro de Investigaciones Apícolas Tropicales, Universidad Nacional, Heredia, Costa Rica; 17 Dep. de Ciências Animais, Universidade Federal Rural do Semi-Arido, Mossoró, RN Brazil; 18 Universidad de Guadalajara, Centro Universitario de la Costa Sur, Autlán, Jalisco México; 19 Departamento de Medicina y Zootecnia de Abejas, Conejos y Organismos Acuáticos, Facultad de Medicina Veterinaria y Zootecnia de la Universidad Nacional Autónoma de México, Ciudad Universitaria, Delegación Coyoacán, 04510 Mexico City, Mexico; 20 Universidade Federal de Sergipe, Campus do Sertão, Departamento de Educação em Ciências Agrárias e da Terra, e Instituto Nacional de Ciência e Tecnologia em Estudos Interdisciplinares e Transdisciplinares em Ecologia e Evolução (INCT-IN-TREE), Mossoró, Brazil; 21 Federación Colombiana de Apicultores y Criadores de Abejas, Bogota, Colombia; 22 Centro Integrado de Pesquisas Biotecnológicas, Campus São Gabriel, Universidade Federal do Pampa (UNIPAMPA), Rua Aluízio Barros Macedo, Br 290, km 423 Bairro Piraí, São Gabriel, RS 97300-000 Brazil; 23 Centro de Biodiversidad y Descubrimiento de Drogas, Instituto de Investigaciones Científicas y Servicios de Alta Tecnología (INDICASAT-AIP), Clayton, 0843-01103 Panamá; 24 Departamento de Zootecnia, Centro de Ciências Agrárias, Universidade Federal do Ceará, Fortaleza, CE 60356-000 Brazil; 25 Instituto de Ecología Regional (IER-CONICET), Tucumán, Argentina; 26 Centro Pyme Adeneu, Agencia de desarrollo económico del Neuquen, Neuquén, Argentina; 27 Department of Biology, University of Puerto Rico Rio Piedras Campus and Institute of Neurobiology, Medical Sciences Campus, San Juan, Puerto Rico; 28 Departamento de Biologia, Faculdade de Filosofia Ciências e Letras de Ribeirão Preto, Universidade de São Paulo, Ribeirão Prêto, SP Brazil; 29 Departamento de Producción Animal, Universidad de Buenos Aires, Facultad de Agronomía, Buenos Aires, Argentina; 30 INTA, Centro de Investigación en Recursos Naturales, Instituto de Recursos Biológicos, Buenos Aires, Argentina; 31 Associação de Proteção às Abelhas Bee or not to Be, Ribeirão Prêto, SP Brazil; 32 Universidad Nacional de Río Negro, Sede Andina, Escuela de Producción Agropecuaria y Tecnología Ambiental, El Bolsón, Argentina; 33 Estación Experimental Agropecuaria INTA Cuenca del Salado, Agencia de Extension Rural Chascomus, Buenos Aires, Argentina; 34 Director de Producción Apícola del Ministerio del Agro y de la Producción de la Provincia de Misiones. Centro de Investigación Apícola y Meliponícola del Instituto Superior del Profesorado en Ciencias Agrarias y Protección Ambiental (PROCAyPA), Misiones, Argentina; 35 Unidad Académica de Animales de Granja, Facultad de Veterinaria, Universidad de la República, Montevideo, Uruguay; 36 Instituto de Innovación para la Producción Agropecuaria y el Desarrollo Sostenible (IPADS) Balcarce (INTA-CONICET), RN 226 km 73.5,7620, Balcarce, Buenos Aires, Argentina; 37 Facultad de Ciencias Agrarias, Universidad Nacional de Mar del Plata (FCA-UNMdP), Ruta 226km 73.5, Balcarce, 7620 Buenos Aires, Argentina; 38 Laboratorio de Investigaciones en Abejas, Departamento de biología, Facultad de Ciencias, Universidad Nacional de Colombia, sede Bogotá, Colombia; 39 Grupo de Investigaciones para la Gestión y Conservación de Servicios Ecosistémicos, Corporación para la Gestión de Servicios Ecosistémicos, Polinización y Abejas-SEPyA, Bogotá D.C., Colombia; 40 Facultad de Zootecnia, Universidad Nacional del Centro del Perú, Av. Mariscal Castilla N° 3909, El Tambo, Huancayo, Perú; 41 Embrapa Recursos Genéticos e Biotecnologia, Parque Estação Biológica, Avenida W5 Norte (Final), Caixa Postal 02372, Brasília, DF 70770-917 Brazil; 42 Centro de Microbiología Básica y Aplicada (CEMIBA), Facultad de Ciencias Veterinarias, Universidad Nacional de La Plata (UNLP) y Consejo Nacional de Investigaciones Científicas y Técnicas, La Plata (CCT-CONICET, La Plata), La Plata, Buenos Aires, Argentina; 43 Centro de Investigaciones Apícolas, Havana, Cuba; 44 Laboratorio de Ecología Química, Facultad de Química, Universidad de la República, Montevideo, Uruguay; 45 Facultad de Ciencias, Iguá 4225, 11400 Montevideo, Uruguay; 46 Instituto de Genética E. A. Favret, Instituto Nacional de Tecnología Agropecuaria (INTA), Consejo Nacional de Investigaciones Científicas y Técnicas (CONICET), Hurlingham, Buenos Aires, Argentina; 47 Sección Apicultura, INIA La Estanzuela, Colonia, Uruguay; 48 Centro de Investigaciones en Abejas (CIABE), Centro Universitario del Sur, Universidad de Guadalajara, Enrique Arreola Silva 883, Cd., Guzman, JAL Mexico; 49 Facultad de Ciencias Veterinarias y Pecuarias, Beeing Company, Departamento Ciencias Universidad de Chile, Avda. Santa Rosa 11315, La Pintana, 882080 Santiago, Chile; 50 Instituto de Biologia, Universidade Federal da Bahia, Campus de Ondina, Rua Barão de Geremoabo s/n, Salvador, BA 40170-210 Brazil; 51 Departamento de Ciencias Agronómicas y Recursos Naturales, Facultad de Ciencias Agropecuarias y Forestales, Universidad de La Frontera, Temuco, Chile

**Keywords:** Beekeeping, Colony loss, Meliponiculture, Monitoring program, Pollinators, South America, Entomology, Ecological epidemiology, Animal physiology, Ecology, Zoology

## Abstract

Over the last quarter century, increasing honey bee colony losses motivated standardized large-scale surveys of managed honey bees (*Apis mellifera*), particularly in Europe and the United States. Here we present the first large-scale standardized survey of colony losses of managed honey bees and stingless bees across Latin America. Overall, 1736 beekeepers and 165 meliponiculturists participated in the 2-year survey (2016–2017 and 2017–2018). On average, 30.4% of honey bee colonies and 39.6% of stingless bee colonies were lost per year across the region. Summer losses were higher than winter losses in stingless bees (30.9% and 22.2%, respectively) but not in honey bees (18.8% and 20.6%, respectively). Colony loss increased with operation size during the summer in both honey bees and stingless bees and decreased with operation size during the winter in stingless bees. Furthermore, losses differed significantly between countries and across years for both beekeepers and meliponiculturists. Overall, winter losses of honey bee colonies in Latin America (20.6%) position this region between Europe (12.5%) and the United States (40.4%). These results highlight the magnitude of bee colony losses occurring in the region and suggest difficulties in maintaining overall colony health and economic survival for beekeepers and meliponiculturists.

## Introduction

Monitoring population status can help understand the causes and consequences of current global changes affecting individual physiology, biological interactions and ecosystem functioning. Specifically, the benefits of large-scale bee monitoring programs allowed researchers to document the ongoing decline of wild bee populations in Europe^1,2^ and in the United States^3^. Wild and managed bees are critical pollinators, essential for the maintenance of biodiversity in natural ecosystems and for agricultural production, increasing the yield and quality of the majority of crops^4–10^. Managed colonies of the Western honey bee, *Apis mellifera*, are commonly used for the pollination of many pollinator-dependent crops^11^. Beyond its ecosystem services, the honey bee has an economic importance for its commercialized hive products, such as honey, propolis and beeswax; supporting thousands of farmers and beekeepers’ families, and representing also a social and cultural value ^5^. Despite the global increase in the number of managed honey bee colonies during several decades^12,13^, recent studies report high rates of colony losses in the United States ^14,15^ and Europe ^16–20^, that constrain substantially the beekeeping activity and threaten crop pollination services^5^.

Concerns about the colony loss of managed honey bees have thus motivated monitoring programs over the last quarter century. The use of national surveys has been expanded to study the health status of honey bee colonies. Among the most notable, the Bee Informed Partnership (*BIP*) has developed since 2007 a national monitoring program in the United States ^21^, while other consortia like the Prevention of honey bee COlony LOSSes (*COLOSS*) have developed theirs in Europe^22,23^. These successful large-scale monitoring programs have in common the establishment of standardized questionnaires and the centralization of data collection and analysis^24^. These large-scale monitoring initiatives contributed substantially to identify risk factors such as flower resource availability^25–28^, beekeeping management^15,17,28–30^ and climate^31–34^. However, our current knowledge of the extent and causes of bee colony losses is mostly based on published studies carried out in the Northern Hemisphere, in particular, in the United States and Europe (but see smaller-scale studies from other regions like China ^35,36^, South Africa^37^, Japan^38^, Canada^39^, and some Latin American countries^40–43^), whereas several countries from the Southern Hemisphere play critical roles as suppliers to the global honey market and the bee-driven crop pollination services^41^.

Latin America (LA) plays an important role in the global food supply. Major food-producing countries (Argentina, Brazil, Chile, Mexico and Uruguay) contribute together about 228.1 million tons of food that is attributable directly to insect pollination, with an economic value of US$ 22.95 billion^9^. In addition, LA has a critical role in the global honey supply, producing 28% of the honey with 7.7 million managed honey bee colonies^13^ and seven LA countries are among the 20 largest producers leading the global honey market^41^. In addition to beekeeping, known as apiculture, the keeping of different species of stingless bees (Meliponini tribe), known as meliponiculture, is an important economic, cultural, social, environmental, and historical practice in the region^44^. The Mayas of the Yucatan Peninsula, northern Guatemala and Belize among other ancient cultures, used this practice more than 1400 years ago; and it is believed to have spread to other civilizations^44^. Currently, meliponiculture is a tool for sustainable development, representing in some cases the source of additional incomes for rural communities and a competitive economic activity in different regions (*e.g.* Brazil^45^ and Mexico^44^). More than 400 species of stingless bees have been identified in LA^45^, from which at least 12% are managed for honey production^46^. Little is known about the rates of colony loss of stingless bees, either naturally or under management. A study in Costa Rica estimated the natural loss of a group of stingless species and found an annual rate of 6.7% excluding predation (human or animal) and 11% including predation^47^.

Despite the key role of the LA region in the supply of bee-pollinated crops and honey, there is a critical lack of surveys, estimates and standardized published data for honey bee and stingless bee colony loss rates. Previous national or regional initiatives launched to estimate colony losses of honey bees in some LA countries found annual loss rates reaching up to 50% in Brazil from 2013 to 2017^42^, 29% in Uruguay from 2013 to 2016^40,41^ and 22% in Argentina from 2010 to 2016^42,48^. These studies suggested an important heterogeneity in the colony loss estimates. However, performing data comparisons between years and countries based on the cited studies is not adequate because they include different survey methods and statistical analyses^41^. Furthermore, to our knowledge, no survey-based estimates of managed stingless bee colony losses have been performed to date.

The heterogeneity in social, cultural, environmental, and political features in LA poses challenges for carrying out standardized monitoring^24^. For instance, beekeeping activity can range from small sized operations as performed by hobbyist producers to professional beekeepers owning thousands of colonies; from the use of traditional practices to new technologies; and from isolated self-trained management to cooperative associations facilitating the access to regional or national training with qualified technical advisors^49^. The variety of ecoregions in LA ranging from arid to tropical climates, and where honey bees and stingless bees are exploited, is worthy of pointing out as an additional challenge for monitoring. Moreover, the socio-economic contexts widely differ among LA countries, which in most cases lack governmental support for beekeepers suffering colony losses. This situation potentially correlates not only with variations in the patterns of bee mortality, but also with different outcomes regarding the continuity of the activity after a significant loss.

Here we present the first large-scale standardized survey of colony loss of managed honey bees and stingless bees in LA, carried out by the Latin American Society for Bee Research (*SOLATINA*^41,50^). This survey was inspired by previous *BIP* and *COLOSS* surveys^31,51^ and adapted to apiculture and meliponiculture activities in LA. We first analyzed the heterogeneity in participants’ profiles and its effect on colony loss, predicting that colony loss can be affected by operation size. Then, we compared colony loss between years and among LA countries in honey bees and stingless bees. We then explored the question of potential interspecific variability in colony loss by comparing colony losses of honey bees and stingless bees in countries and years where data for both beekeeping practices were reported. Finally, we assessed the overall magnitude of honey bee colony losses in LA in a broader geographic context by comparing our data with published data for the same years in the United States and Europe.

## Results

### Participants’ profile effect on operation size

**Figure 1:**
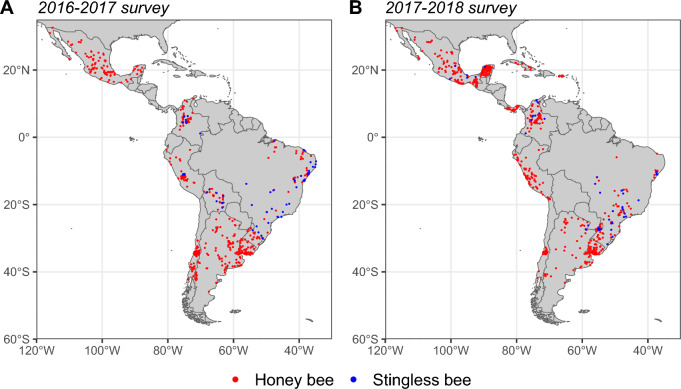
Spatial distribution of the data collection for (**A**) the 2016–2017 survey and (**B**) the 2017–2018 survey over Latin America. The colors show the response from beekeepers (in red) and meliponiculturists (in blue).

A total of 1901 participants with complete responses were recorded for this two-year survey (n = 902 in 2016–2017 and n = 999 in 2017–2018) across a large extent of the LA region (Fig. 1), of whom 1736 were honey bee beekeepers (n = 809 in 2016–2017 and n = 927 in 2017–2018) and 165 were meliponiculturists (n = 93 in 2016–2017 and n = 72 in 2017–2018). The overall number of participants in the survey varied across countries and years for honey bees (Table 1) and stingless bees (Table 2). Importantly, the representativeness of the data ranged between 0.9 and 31.7% of the registered colonies for each country (Tables 1, 2). Among beekeepers, the size of the operation (log-transformed number of colonies) was significantly different for professional (197 [110–354] honey bee colonies, mean [95% CI]), semi-professional (61 [37–99] honey bee colonies), and hobbyist beekeepers (17 [9–30] honey bee colonies; Fig. S1, Table S1). We found a significant difference between beekeepers and meliponiculturists in operation size, with fewer colonies owned by meliponiculturists (Table S1), and a similar significant trend between professional (143 [93–218] stingless bee colonies), semi-professional (26 [20–36] stingless bee colonies), and hobbyist meliponiculturists (9 [6–14] stingless bee colonies; Fig. S1). Interestingly, we found that operation size was affected by country, but not by the interaction between operation type and country (Fig. S1, Table S1), meaning that the same operation type trend is observed across countries.

**Table 1:** Total colony losses of honey bees predicted per country and year with a 95% Confidence Interval (based on GLMs).

Year	% colonies	N	No. colonies	% Summer loss (95% CI)	N	No. colonies	% Winter loss (95% CI)	N	No. colonies	% Annual loss (95% CI)
Argentina										
2016–2017	1.76	135	50,805	22.1 (18.6–26)	121	46,778	20.9 (17.2–25.3)	111	44,578	34.8 (30.3–39.7)
2017–2018	1.58	107	41,731	13.8 (10.9–17.3)	110	46,610	11.9 (9.2–15.4)	91	40,499	21.4 (17.5–25.9)
Bolivia										
2016–2017	–	76	2266	27.1 (14.5–44.8)	67	2246	20.5 (10–37.5)	61	1901	36.3 (21.4–54.4)
Brazil										
2016–2017	0.89	114	8639	27 (20.2–35.2)	112	5085	23.2 (14.7–34.7)	95	4719	44.7 (34–56)
2017–2018	2.63	88	25,345	13 (10–16.7)	66	20,765	31.9 (26.2–38.2)	60	19,384	27 (21.8–32.8)
Chile										
2016–2017	6.42	120	27,877	28.7 (24.5–33.4)	90	17,487	21 (15.9–27.2)	74	16,511	42.1 (36–48.5)
2017–2018	6.08	97	21,595	15.3 (11.9–19.6)	97	25,101	26.1 (21.5–31.4)	85	19,507	25.6 (20.9–30.9)
Colombia										
2016–2017	7.5	41	8005	29.6 (22.1–38.4)	38	8335	21.9 (14.8–31.3)	37	7910	42.8 (34.3–51.8)
2017–2018	30.46	70	30,827	40.2 (33.9–46.8)	63	31,882	28.5 (22.8–35.1)	52	28,384	52.5 (45.8–59.1)
Cuba										
2017–2018	3.77	14	6224	22.6 (15.7–31.3)	13	5142	7 (2.9–15.8)	12	4140	18.8 (11.2–29.8)
Mexico										
2016–2017	1.1	86	16,963	3.4 (1.8–6.3)	91	20,110	20.9 (16.5–26)	83	16,644	17.3 (13.2–22.4)
2017–2018	1.42	226	29,590	6.1 (4.3–8.6)	227	30,623	11.6 (8.9–15.1)	220	29,308	15.2 (12–18.9)
Panama										
2017–2018	–	25	1768	8.2 (2.3–25.4)	28	1969	12 (4.5–28.5)	25	1768	17.1 (7.2–35.2)
Peru										
2016–2017	–	66	2920	12.3 (5.6–24.8)	33	8374	23.8 (16.5–33.1)	30	755	12.7 (2.6–44.4)
2017–2018	–	94	16,722	31.4 (25.6–37.8)	105	19,148	23.2 (18.1–29.2)	70	13,446	41.5 (34.6–48.7)
Puerto Rico										
2017–2018	31.65	10	575	7.8 (0.6–53.5)	10	810	15.5 (3.9–45.1)	9	460	27.9 (7.2–65.9)
Uruguay										
2016–2017	7.52	109	43,888	14.1 (11.6–17.1)	43	14,781	29.2 (23.1–36.2)	42	14,629	38.2 (31.8–45)
2017–2018	6.12	88	29,108	16.3 (13.1–20)	92	30,398	22 (18.1–26.5)	79	25,487	32 (27.4–37.1)

*N* is the number of respondents. *No. colonies* is the sum of colonies per period (i.e. summer, winter, annual). *% Colonies* is the proportion of the colonies included in the study regarding the total number of managed colonies registered in the country based on the FAO dataset (FAOSTAT 2023).

**Table 2:** Total colony losses of stingless bees predicted per country and year with a 95% Confidence Interval (based on GLMs).

Year	N	No. colonies	% Summer loss (95% CI)	N	No. colonies	% Winter loss (95% CI)	N	No. colonies	% Annual loss (95% CI)
Argentina									
2017–2018	22	398	42.1 (13.6–77.1)	21	354	15 (1.9–62)	20	335	42.9 (14.2–77.4)
Bolivia									
2016–2017	12	187	48.9 (8–91.3)	11	176	44.8 (7.5–89.1)	10	168	65 (16.8–94.4)
Brazil									
2016–2017	51	2044	36.8 (23.2–52.9)	48	1650	15.3 (6–33.5)	44	1386	42.8 (26.3–61.2)
2017–2018	31	1323	28.1 (14.4–47.6)	24	658	14.9 (3.6–45)	23	648	30.4 (12–58.5)
Colombia									
2016–2017	14	550	18.7 (4.6–52.2)	15	599	8.7 (1.2–42.5)	12	537	22.3 (6.5–54)
2017–2018	10	573	32.9 (12.6–62.4)	9	311	5.3 (0.1–70.9)	8	293	31.7 (7.7–72.1)
Mexico									
2017–2018	6	262	39.9 (6.1–87.1)	6	262	61 (19.6–90.9)	6	262	67.3 (25.1–92.6)
Peru									
2016–2017	8	51	0 (0–100)	7	43	12.4 (0.1–97.4)	7	43	14.7 (0–98.6)

*N* is the number of respondents*. No. colonies* is the sum of colonies per period (i.e. summer, winter, annual).

### Honey bee colony loss

**Figure 2:**
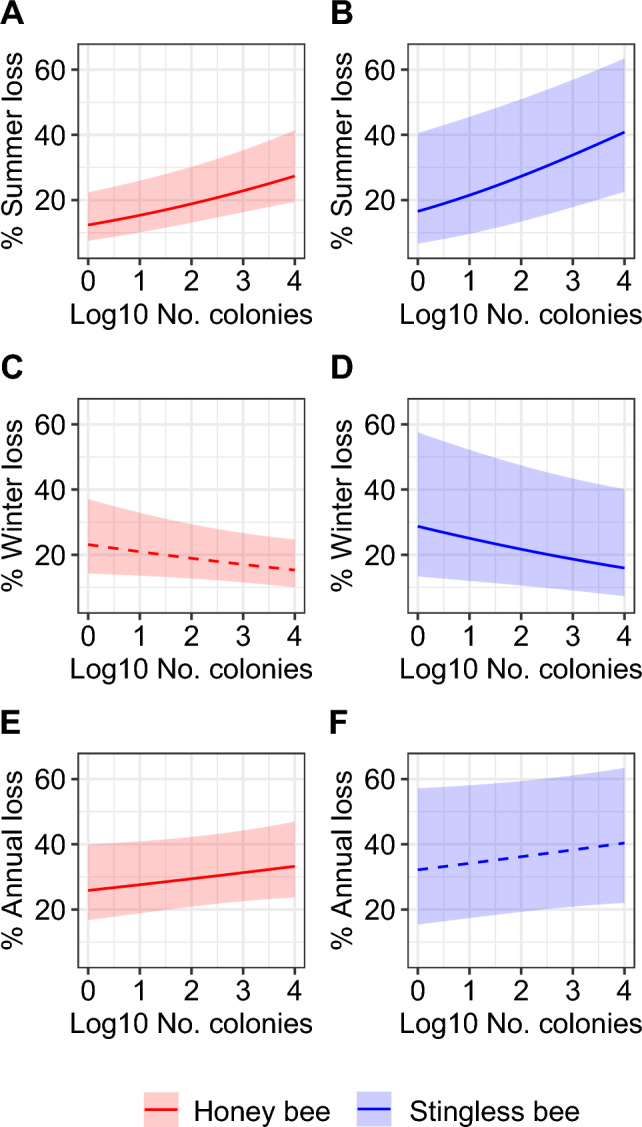
Operation size (log-transformed number of colonies) effects on colony loss in (**A**, **B**) summer, (**C**, **D**) winter, and (**E**, **F**) annual periods in Latin America. Honey bees are presented in red (**A**, **C**, **E**) and stingless bees in blue (**B**, **D**, **F**). Thick lines show the GLM predictions with shaded areas indicating the 95% CI. These lines are dashed if they are non-significant.

**Figure 3:**
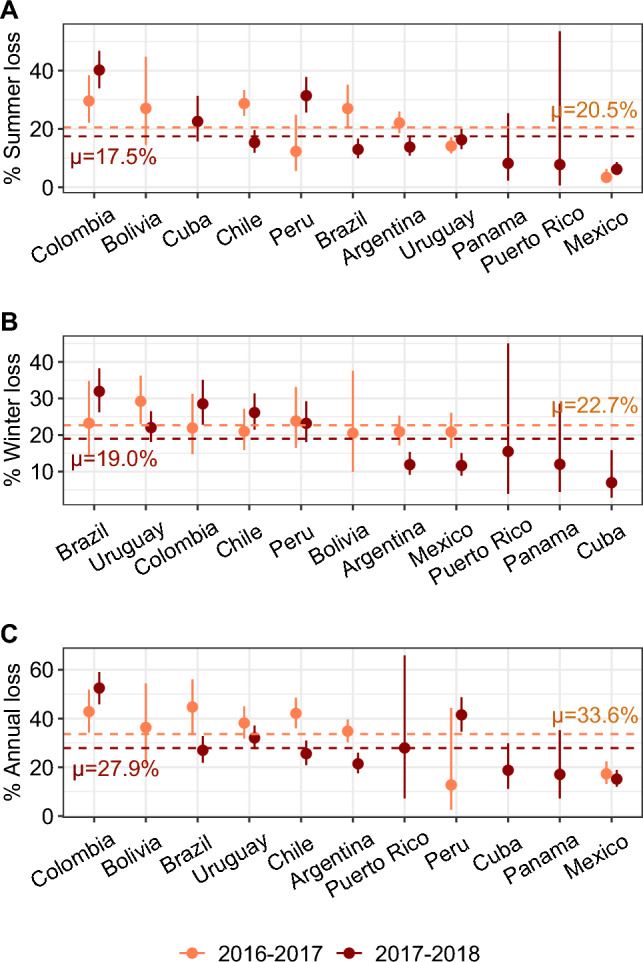
Honey bee colony loss for (**A**) summer, (**B**) winter, and (**C**) annual periods in Latin America. Dots represent the predicted total loss per country and year, with thick lines indicating the 95% CI (based on GLM predictions). Countries are ordered by decreasing loss values. The colors distinguish the two years of the data collection (2016–2017 in orange and 2017–2018 in brown). Horizontal dashed lines represent the average value for Latin America with different colors when the year effect was significant.

We found that summer colony loss and annual colony loss increased with operation size in honey bees (Fig. 2, Table 3). In contrast, we observed a negative pattern of operation size on winter colony loss, although not significant (Table 3). Overall, average annual colony loss ranged from 16.2% (95% CI: 12.6–20.6%) in Mexico to 47.7% (95% CI: 40.0–55.5%) in Colombia (Fig. 3). We found significant country effects for summer (average of 18.8%, 95% CI: 13.7–27.9%), winter (20.6%, 95% CI: 14.7–29.5%) and annual colony losses (30.4%, 95% CI: 22.7–41.5%) (Fig. 3, Table 3). Summer, winter and annual colony losses significantly differed between years, with higher losses in 2016–2017 than in 2017–2018 (Fig. 3, Table 3). Furthermore, we found significant interacting effects between countries and years for summer, winter and annual losses (Table 3), meaning that the year effect was not systematically in the same direction for all countries. For instance, annual loss was higher in 2016–2017 than 2017–2018 for Brazil, but higher in 2017–2018 than 2016–2017 for Peru (Fig. 3, Table 1).

**Table 3:** Summary of the Generalized Linear Models performed to evaluate the effects of country, year, operation size (log-transformed number of colonies) and the interaction between country and year on colony loss of honey bees in Latin America.

Parameter	Deviance	*F* value	*P* value
Summer			
** Country**	**19,557.8**	**32.3**	** < 0.001**
** Year**	**402.5**	**6.7**	**0.010**
** Operation size**	**1723.9**	**28.5**	** < 0.001**
** Country × Year**	**3686.6**	**10.2**	** < 0.001**
Winter			
** Country**	**4692.3**	**8.5**	** < 0.001**
** Year**	**601.3**	**10.9**	** < 0.001**
Operation size	127.3	2.3	0.129
** Country × Year**	**1677.5**	**5.1**	** < 0.001**
Annual			
** Country**	**14,171.8**	**22.8**	** < 0.001**
** Year**	**1842.0**	**29.6**	** < 0.001**
** Operation size**	**387.3**	**6.2**	**0.012**
** Country × Year**	**2235.5**	**6.0**	** < 0.001**

Bold lines indicate significant differences (*P* < 0.05).

### Stingless bee colony loss

**Figure 4:**
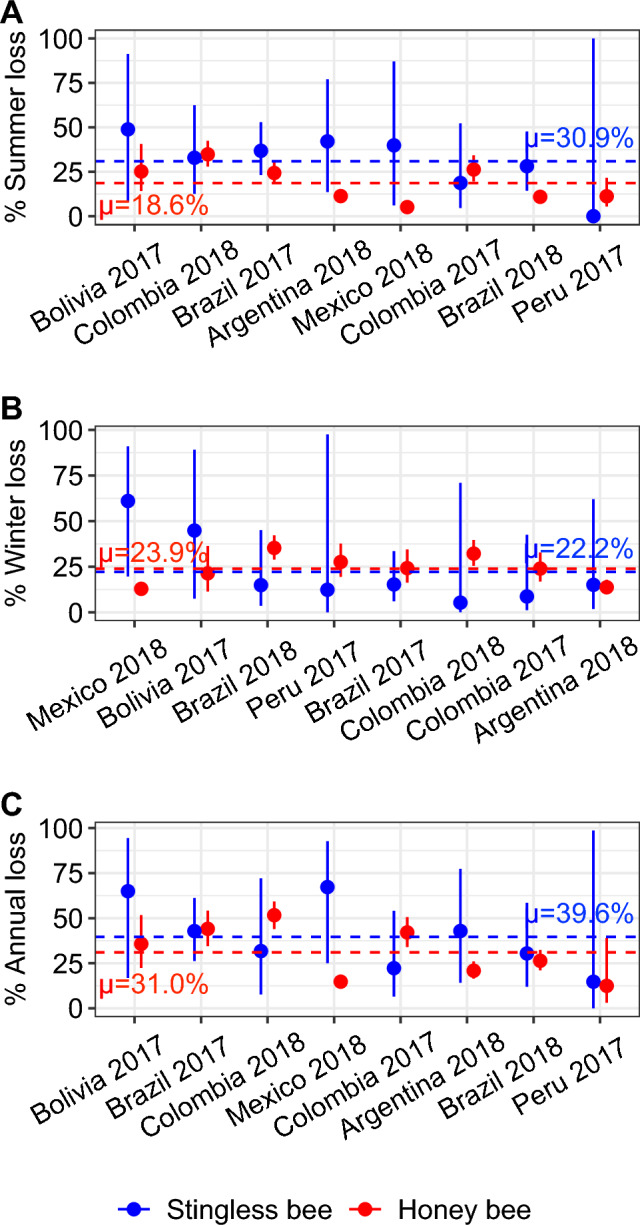
Stingless bee colony loss comparison with honey bees for (**A**) summer, (**B**) winter, and (**C**) annual periods in Latin America. Dots represent the predicted total loss per country and year, with thick lines indicating the 95% CI (based on GLM predictions). Countries are ordered by decreasing loss values. Horizontal dashed lines represent the average value of stingless bee loss (in blue) and honey bee loss (in red) for Latin America.

We found that colony loss increased with operation size during summer and decreased during winter in stingless bees, but we found no effect of operation size on annual loss (Fig. 2, Table 4). Overall, annual colony loss ranged from 14.7% (95% CI: 0.0–98.6%) in Peru in 2016–2017 to 65.0% (95% CI: 16.8–94.4%) in Bolivia in 2016–2017 (Fig. 4). We found significant Country-Year effects (the combination of country identity and year of the survey as a unique fixed factor) for summer (average of 30.9%, 95% CI: 10.3–71.3%), winter (22.2%, 95% CI: 5.0–66.4%) and annual colony losses (39.6%, 95% CI: 13.6–76.1%) (Fig. 4, Table 4). Interestingly, when pooling data from beekeepers per year and country and comparing it with pooled data from meliponiculturists in the same way, we found no difference between stingless bees and honey bees (i.e. bee type factor) in summer, winter and annual losses, and no significant interaction between Country-Year and bee type on colony losses (Table 4).

**Table 4:** Summary of the Generalized Linear Models performed to evaluate the effects of Country-Year, bee type, operation size (log-transformed number of colonies) and the interaction between Country-Year and bee type on colony loss in Latin America.

Parameter	Deviance	*F* value	*P* value
Summer			
** Country-Year**	**14,622.7**	**42.2**	** < 0.001**
Bee type	123.4	2.5	0.115
** Operation size**	**1202.3**	**24.3**	** < 0.001**
Country-Year × Bee type	444.0	1.3	0.257
Winter			
** Country-Year**	**4765.3**	**16.2**	** < 0.001**
Bee type	28.4	0.7	0.411
** Operation size**	**327.7**	**7.8**	**0.005**
Country-Year × Bee type	515.7	1.8	0.094
Annual			
** Country-Year**	**13,103.7**	**38.4**	** < 0.001**
Bee type	2.4	0.05	0.823
Operation size	115.1	2.4	0.125
Country-Year × Bee type	625.7	1.8	0.078

Country-Year combines the country identity and the year of the survey as a unique factor (*e.g.* Bolivia 2017). Bee types include honey bees and stingless bees. Bold lines indicate significant differences (*P* < 0.05).

### Magnitude of honey bee colony losses in LA compared to the United States and Europe

**Figure 5:**
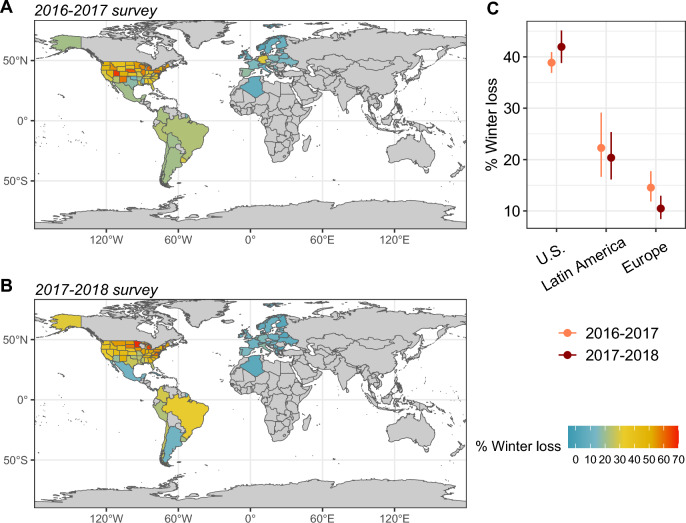
Comparison of winter colony loss of honey bees among large-scale monitoring initiatives including Latin America (the present *SOLATINA* initiative), the United States (the *BIP* initiative) and Europe (the *COLOSS* initiative, that also includes non-European countries such as Algeria). (**A**) Winter loss over the 2016–2017 survey and (**B**) over the 2017–2018 survey. The winter period represents October 1st to March 31st in the Northern Hemisphere and April 1st to September 30th in the Southern Hemisphere. (**C**) Predicted total winter loss of honey bees per large-scale monitoring initiative and year, with thick lines indicating the 95% CI (based on GLM predictions). Large-scale monitoring initiatives are ordered by decreasing loss values. The colors distinguish the two years of the data collection (2016–2017 in orange and 2017–2018 in brown).

By comparing large-scale monitoring initiative data, we found that the mean winter colony loss for the same two years in LA (average of 21.3%, 95% CI: 16.4–27.2%) was significantly higher than Europe (12.5%, 95% CI: 10.1–15.4%) and significantly lower than the United States (40.4%, 95% CI: 37.8–43.0%), placing LA losses between these two regions (Fig. 5). We found no effect of the year on winter colony loss, meaning that colony losses were stable across years (Table S2). Interestingly, we found a significant interacting effect between the large-scale monitoring initiative and years, meaning that the year effect was not systematically in the same direction for all large-scale monitoring initiatives. For instance, winter loss was higher in 2016–2017 than 2017–2018 for Europe, but higher in 2017–2018 than 2016–2017 for the United States (Fig. 5).

## Discussion

Over the last 20 years, large-scale monitoring initiatives have been developed to estimate honey bee colony loss in different regions of the world, of which the LA region was poorly documented ^24^. These surveys highlighted generalization of colony losses between 20 and 40% of the livestock of beekeepers each year in the United States^14,15,21,29,32,52,53^ and Europe^16–18,22,28^, leading to important concerns on the sustainability of the beekeeping activity and the crop pollination services. In this study, we present the results of the first large-scale, standardized survey of honey bee and stingless bee colony losses in LA. Based on two years of data collection, our results suggest difficulties for both beekeepers and meliponiculturists in LA. We considered that two years of data were not sufficient to infer temporal patterns. Furthermore, we cannot attribute any resilience to effective beekeeping management strategies as we have not tested this hypothesis with this dataset. Alternatively, we interpret the interannual difference in loss rate as illustrating the natural variability of the system and the need for long-term monitoring to capture temporal trends and test the influence of beekeeping strategies on them.

Overall, about one-third of honey bee and stingless bee colonies were lost on average per year in LA. Although these losses do not imply a decline in the number of hives at national or regional level^12^, as beekeepers can compensate for them by buying new colonies or dividing surviving colonies, this may represent an additional cost to beekeepers and the sustainability of beekeeping. Results are particularly alarming in countries such as Colombia, Bolivia and Brazil, with records of high colony loss rates (above 30% annual losses) for both honey bees and stingless bees. Conversely, other LA countries such as Mexico, Cuba and Panama have relatively low colony loss rates (below 20% annual loss), illustrating the heterogeneity of situations across LA. Conducting questionnaire-based surveys in developing countries is much more complex than in developed countries because it requires face-to-face interviews, which limits the collection of quantitative data ^22^. Nevertheless, our survey included a number of answers similar to, or even larger than, other studies previously published in countries of the region (e.g., Argentina^48^, Brazil ^42^, Mexico^16,17,28,54–56^, Uruguay ^40^). In order to avoid over-interpretation of the results, we only considered data from countries where our survey covered more than 1% of the representativeness (i.e. we excluded countries where the number of answers was lower, e.g. Costa Rica, Ecuador, Honduras, Paraguay, and Venezuela), in some cases reaching 30%, which is an accepted measure for proper estimation of colony losses^16,17,28,40,42,48,54–56^.

We tested whether operation size could affect colony loss in LA as observed in the United States^15,29,30,57,58^ and Europe^17,59^, and we found that operation size increased summer loss in both honey bees and stingless bees. Since in the United States, summer colony loss by large beekeepers has been attributed to migration of the operation for honey production or commercial crop pollination^15,29,30^, we hypothesize those practices as potential drivers of honey bee colony losses in LA. However, these hypotheses about migration or pollination service effects need to be formally tested. Furthermore, we found the same significant trend with stingless bees. Migration of stingless bee colonies is not a common practice and if performed, it only involves a few stingless bee species. Nevertheless, other management practices could impact the colony health and survival of stingless bees in a different way than for honey bees. During summer, the impact of the temperature and humidity may disturb the homeostasis of the colony since different models of rational hive models used in meliponiculture are commonly adapted to the vast diversity of species ^60^. However, our understanding on the advantages of each type of hive in regards to biological parameters is scarce ^61^. Furthermore, the summer period also increases the activity of predators or robbers, sometimes coupled with the exposure to fumigation to control insects vectoring diseases in the hives. Another hypothesis is the competition for food during the summer period with *A. mellifera* or even between stingless bee colonies in an environment with a high density of colonies^62,63^ and limitation in flower resources^64^. However, competition between *A. mellifera* and stingless bees has not been systematically observed in LA^65–67^. Overall, we found that colony loss rates for stingless bees were higher (ranging from 14 to 65%) than the naturally occurring rates found in Costa Rica (6.7% and 11% ^47^). Comparing these rates may highlight the challenge for meliponiculturists to keep their colonies alive and the need to standardise best management practices for these species. However, this comparison should be taken with caution as it is important to note that the sample size of natural populations was small (i.e. 192 colonies over 4 years^47^).

We found that winter colony loss decreased with operation size in stingless bees, but not in honey bees although a negative pattern (not significant) was observed. These results also agree with the findings in the United States^15,29,30,57,58^ and Europe^17,59^. One hypothesis to explain the non-significant pattern with honey bees could be that the large heterogeneity of participants’ profiles would mitigate the operation size effect. For instance, professional beekeepers in Peru own fewer colonies (mean = 119 colonies) than semi-professional beekeepers in Uruguay (mean = 220 colonies), illustrating the large variety of beekeeper profiles and situations across the different LA countries. The operation size does not integrate all practices and contexts of beekeeping and future studies should consider multiple parameters of beekeeping management practices^68,69^.

Winter is a major challenge for bee colonies in temperate regions, as colonies must survive months with low temperatures, confinement in the hive and lack of pollen and nectar intake. Furthermore, in some temperate regions, the temperature still allows foraging flights and laying activity of the queen and therefore the possibility of parasite reproduction in preimaginal stages, such as *Varroa* mites. It is expected that weak colonies, with a low bee population and/or a lack of adequate reserves, will not survive these conditions^70^. Thus, winter colony losses are expected to be higher than summer losses. However, in several LA countries (*e.g.* Argentina, Bolivia, Colombia), similar or even higher loss rates were found in the summer compared to the winter in honey bees. Similar results were observed in the United States, with summer losses reaching or even exceeding winter losses^14,15,21,29,32,52,53^. Moreover, in South Africa where only summer losses were estimated, honey bee colony loss reached up to 46% ^37^. We also observed higher loss rates in summer compared to winter in stingless bees for several LA countries (Argentina, Bolivia, Brazil and Colombia). This high level of summer losses could be associated with the operation size effect as discussed before, but also with other abiotic factors that are known to impact bee health and survival, such as pesticide exposures^42,53,71^, flower resource availability^25–28^ and climate^31–34^. Biotic factors can also be linked with summer colony loss^72–74^, and overall, the combination of all these factors is a hypothesis that should be investigated in future research^75,76^. One point that needs to be clarified is that we estimated colony loss in three fixed periods: from October 1st to March 31st, from April 1st to September 30th, and from October 1st of one year, to September 30th of the next year. Following previous published studies^15,16,24,40,41,54,55^, we allocated these periods to summer, winter and annual losses, respectively, with distinctions between the North and South hemisphere locations of the countries. This method facilitated the comparison of colony loss between LA and other regions of the world (*e.g.* the United States and Europe). However, it is important to notice that LA includes a large variety of climates and the generalization of the terms “summer” and “winter” could not fit rigorously with the entire region. Part of the LA region (e.g. areas within a country or across multiple countries) could be distinguished between dry or rainy seasons instead of winter or summer periods, although this specificity would limit comparisons across regions.

Overall, this study shows the results of the first large-scale monitoring initiative in LA and reveals an alarming situation for honey bees and native stingless bees in the region. Comparing honey bee colony losses between large-scale monitoring initiatives from the perspective of cross-continental analysis, we found that winter losses in LA places between the United States and Europe trends. This result confirms that honey bee colony losses are a global concern, and Latin America is not exempted. Although direct comparisons between datasets should be made with caution, since different sample sizes and methods were used for data collection, the results highlight the alarming situation of the LA. Similarly to beekeepers, meliponiculturists are suffering a high rate of colony loss in LA, and are also facing some other challenges in the sustained development of meliponiculture as an increasingly expanding practice in the region. These challenges include the acquisition of colonies through non-extractive methods (which are becoming less common but still persist) and the regulation of trade and movement of colonies to non-native locations, which has been observed to potentially contribute to the loss of colonies of native bees^77^. Moreover, the management and control of the major Phoridae fly pests impacts the survival of managed stingless bees in central America^78^. Besides the importance of those native bees in the maintenance of biodiversity in natural ecosystems, and the improvement of agricultural production, meliponiculture is a tool for sustainable development, representing additional incomes, food and medicine for rural communities. It is necessary to promote the sustainable growth of this activity, considering local and traditional knowledge, and it is also important to better understand the biology and diversity of the species involved by means of accurate scientific approaches. This study also highlights the need (1) to coordinate among individual survey initiatives^41,79^, (2) to standardize methods^22,52^, and (3) to consider seasonal (e.g. summer/winter, dry/rainy seasons) and annual losses, in order to improve the effectiveness of monitoring initiative, to enable international comparisons and enhance bee health.

This study help understanding beekeeping challenges that occur in LA in order to foster research on how biotic and abiotic risk factors potentially involved in colony losses, such as pests and pathogens^33,43,78^, flower resource availability ^25–28^, beekeeping management^15,17,29,30,57–59^, pesticide exposures^53,71,80^, and climate^31–34,81^, could affect honey bee and stingless bees in the region.

## Methods

### Survey of bee colony loss in Latin America

In October 2017, we established a standardized questionnaire to record bee colony losses in LA (Section S1), partially inspired by surveys that have already proven to be effective in other regions, specifically, those developed by *BIP*^32^ and *COLOSS*^51^. We first adapted the questionnaire to LA conditions. For this we split the year into two periods of six months: from April 1st to September 30th, and from October 1st to March 31st. Because LA is placed on both hemispheres, we distinguished countries from the Northern Hemisphere (*e.g.* Cuba, Mexico, Panama and Puerto Rico) to those from Southern Hemisphere (*e.g.* Argentina, Bolivia, Chile, Peru, and Uruguay). Based on previous papers on colony losses in the Americas, we considered winter in North America from October 1st to March 31st and summer from April 1st to September 30th^15–17,54,55^, while we considered winter in South America from April 1st to September 30th and summer from October 1st to March 31st^24,40,41^. This method facilitated the comparison of colony loss between LA and other regions of the world (*e.g.* the United States and Europe). Moreover, we created a specific questionnaire for meliponiculture, an activity different from beekeeping, with dozens of native bee species kept by meliponiculturists, including *e.g. Melipona beecheii, Scaptotrigona tubiba, Trigona angustula*^45,46,82,83^.

The questionnaire, available in Spanish and Portuguese, was distributed through regional coordinators in 11 countries, including: Argentina, Bolivia, Brazil, Chile, Colombia, Cuba, Mexico, Panama, Peru, Puerto Rico and Uruguay. These coordinators worked with local beekeeping associations or beekeeping networks, national beekeeping magazines, universities and research institutes. They promoted the online form of the questionnaire using e-mail, social networks (Facebook) and WhatsApp application, distributed the printed form during beekeeping trainings and workshops, and also conducted face-to-face or phone interviews to improve the coverage and representativeness, as recommended for the LA region^24^. Each regional coordinator started the spread of the survey and the face-to-face interviews in October until March each survey year. Information for the 2016–2017 questionnaire was collected from October 2017 until March 2018, while information for 2017–2018 was collected from October 2018 until March 2019.

Each participant (i.e. a beekeeper or meliponiculturist) was invited to answer specific questions about the participant profile, location of the main operation and number of colonies (Section S1). We considered the operation size to be the annual number of colonies owned by the participant. Participants’ profiles were defined as follows: “professional” is a participant whose income comes exclusively from beekeeping; “semi-professional” has several sources of income, including beekeeping; and “hobbyist” does not practice beekeeping for a monetary reward (including traditional meliponiculture). Once received, completed survey responses were transcribed into an Excel sheet and anonymized for further analysis. All methods were carried out in accordance with relevant guidelines. Each participant was advertised of the procedure for anonymization and the possibility to publish the anonymized data. The informed consent was obtained from all participants orally to the coordinators during face-to-face or phone interviews, or by default when the participant voluntarily response to the online form. Given that the interview questions did not request sensitive personal information and respondents were interviewed in their professional roles, and following adherence to the principles of data minimization and purpose limitation during collection, no ethics review was required. All experimental protocols were approved by the SOLATINA’s scientific committee.

### Assessing bee colony loss

To avoid unintentional or willful errors in mortality rate registration, the colony loss was not directly asked to the beekeepers^22^. Conversely, each participant was invited to register the number of colonies alive at different times of the year, as well as the number of colonies given or sold and the number of colonies received or bought during each period, allowing the computation of periodical loss rates (*e.g.* summer, winter and annual losses). Following a published technique^29^, we then calculated the number of colonies alive and lost for each period (summer, winter and annual) as follows:

1 $${\text{No}}. \, \;{\text{colonies }}\;{\text{alive}}\, = \,a + b - c$$

2 $${\text{No}}. \, \;{\text{colonies }}\;{\text{lost}}\, = \,a + b - c - d$$

*a* = Number of colonies alive on the starting date of the period. *b* = Number of colonies made, received or bought during the period. *c* = Number of colonies given or sold during the period. *d* = Number of colonies alive on the ending date of the period.

It is worth noting that “lost colony” was applied for dead or depopulated colonies that were not viable to continue under productive management. Unfortunately, not all beekeepers answered all the questions, thus the sample size differed among summer, winter and annual colony losses. To assess the representativeness of the data, we computed the proportion of the colonies included in the study regarding the number of managed honey bee colonies registered in each country and year based on the Food and Agriculture Organization (FAO) of the United Nations ^13^. Note that no data on managed honey bee colonies was available in the FAO data for Bolivia, Panama, nor Peru. Moreover, FAO data focuses on honey bees and no such information was available for stingless bees.

### Magnitude of colony losses in LA compared to the United States and Europe

In order to compare the winter colony loss of honey bees occurring in LA with other parts of the world, we considered published data from two other large-scale monitoring initiatives from the United States (the *BIP* initiative) and Europe (the *COLOSS* initiative, which also includes non-European countries such as Algeria and Mexico). For the analysis, we did not consider *COLOSS* data for Mexico given that the region was covered by the *SOLATINA*’s initiative. Data for the United States were available at the *BIP* website^84^ and were recently published^15^. Data for Europe were published in 2018 for the 2016–2017 survey^56^ and in 2019 for the 2017–2018 survey^28^ . The winter period represents October 1st to March 31st in the Northern Hemisphere and April 1st to September 30th in the Southern Hemisphere. The definition of colony losses in these two large-scale monitoring initiatives is slightly different. For example, in the BIP initiative, only colonies that have died or disappeared are considered losses, whereas in the COLOSS initiative, colonies that are alive but have insolvent queen problems are also considered losses. For our analysis, we used only the COLOSS data for losses of colonies that died or disappeared for comparison with the other regions.

### Statistical analyses

All analyses were performed using the statistical program R version 3.6.2^85^.

#### Participants’ profile effect on operation size

A Linear Model (LM) was used to compare the operation size (log-transformed number of colonies, response variable) between participants’ profiles (i.e. “hobbyist”, “semi-professional”, “professional”, fixed factor). The model also included other fixed factors such as country (the country of the participant), bee type (beekeeper or meliponiculturist), year (i.e. the 2016–2017 survey or the 2017–2018 survey), and the interaction between participants’ profiles and country.

#### Honey bee colony loss

Generalized Linear Models (GLMs) with a quasibinomial distribution of errors were used to analyze the effects of country (fixed factor), year (fixed factor), operation size (log-transformed number of colonies, fixed factor) and the interaction between country and year on honey bee colony losses (summer, winter and annual periods, response variables). Colony loss was modeled as a binomial response by comparing the number of alive and lost colonies per beekeeper (i.e. the statistical unit). Three models were built corresponding to summer, winter and annual losses.

#### Stingless bee colony loss

In order to analyze whether meliponiculturists suffer similar or different losses in their activities than beekeepers, we focused on years and countries for which we collected data from both beekeepers and meliponiculturists. GLMs with a quasibinomial distribution of errors were used to analyze the effects of Country-Year (the combination of country identity and year of the survey as a unique fixed factor, *e.g.* Bolivia 2017), bee type (i.e. stingless bee or honey bee, fixed factor), operation size (log-transformed number of colonies, fixed factor) and the interaction between Country-Year and bee type on colony losses (summer, winter and annual, response variables). Colony loss was modeled as a binomial response by comparing the number of alive and lost colonies per beekeeper/meliponiculturist (i.e. the statistical unit). Three models were built corresponding to summer, winter and annual losses.

#### Magnitude of honey bee colony loss in LA compared to the United States and Europe

A GLM with a quasibinomial distribution of errors was used to analyze the effects of large-scale monitoring initiatives (i.e. the present *SOLATINA* initiative for Latin America, the *BIP* initiative for the United States, and the *COLOSS* initiative for Europe, fixed factor), year (fixed factor), and the interaction between large-scale monitoring initiative and year on the winter colony loss of honey bees (response variable). Colony loss was modeled as a binomial response by comparing the number of alive and lost colonies per country (or state for U.S., the statistical unit). We considered the total number of colonies for the alive colonies and computed the lost colonies considering the percentage of colony loss.

For all the statistics, model residuals were extracted and inspected against fitted values (residuals versus fitted plot and normal Q–Q plot) to ensure residual normality and homoscedasticity assumptions were fulfilled. The significance level for the statistical tests was set at 5% for the probability of rejecting the true null hypothesis.

### Supplementary Information

Supplementary Information.

## Data Availability

Data available from the figshare repository 10.6084/m9.figshare.23999775.v1^86^.
