# Publisher Correction: First large-scale study reveals important losses of managed honey bee and stingless bee colonies in Latin America

**DOI:** 10.1038/s41598-024-64759-1

**Published:** 2024-06-18

**Authors:** Fabrice Requier, Malena Sibaja Leyton, Carolina L. Morales, Lucas A. Garibaldi, Agostina Giacobino, Martin Pablo Porrini, Juan Manuel Rosso-Londoño, Rodrigo A. Velarde, Andrea Aignasse, Patricia Aldea-Sánchez, Mariana Laura Allasino, Daniela Arredondo, Carina Audisio, Natalia Bulacio Cagnolo, Marina Basualdo, Belén Branchiccela, Rafael A. Calderón, Loreley Castelli, Dayson Castilhos, Francisca Contreras Escareño, Adriana Correa-Benítez, Fabiana Oliveira da Silva, Diego Silva Garnica, Grecia de Groot, Andres Delgado-Cañedo, Hermógenes Fernández-Marín, Breno M. Freitas, Alberto Galindo-Cardona, Nancy Garcia, Paula M. Garrido, Tugrul Giray, Lionel Segui Gonçalves, Lucas Landi, Daniel Malusá Gonçalves, Silvia Inés Martinez, Pablo Joaquín Moja, Ana Molineri, Pablo Fernando Müller, Enrique Nogueira, Adriana Pacini, María Alejandra Palacio, Guiomar Nates Parra, Alejandro Parra-H, Kátia Peres Gramacho, Eleazar Pérez Castro, Carmen Sílvia Soares Pires, Francisco J. Reynaldi, Anais Rodríguez Luis, Carmen Rossini, Milton Sánchez Armijos, Estela Santos, Alejandra Scannapieco, Yamandú Mendoza Spina, José María Tapia González, Andrés Marcelo Vargas Fernández, Blandina Felipe Viana, Lorena Vieli, Carlos Ariel Yadró García, Karina Antúnez

**Affiliations:** 1 Université Paris-Saclay, CNRS, IRD, UMR Évolution, Génomes, Comportement et Écologie, 91198 Gif-sur-Yvette, France; 2 Sociedad Latinoamericana de Investigación en Abejas (SOLATINA), Montevideo, Uruguay; 3 Grupo Ecología de la Polinización, INIBIOMA (CONICET-Universidad Nacional del Comahue), Quintral 1250, Bariloche, Río Negro Argentina; 4 Universidad Nacional de Río Negro, Instituto de Investigaciones en Recursos Naturales, Agroecología y Desarrollo Rural, Bariloche, Río Negro Argentina; 5 Consejo Nacional de Investigaciones Científicas y Técnicas, Instituto de Investigaciones en Recursos Naturales, Agroecología y Desarrollo Rural, Bariloche, Río Negro Argentina; 6 Instituto de Investigación de La Cadena Láctea (INTA-CONICET), Estación Experimental Agropecuaria- Rafaela, Ruta 34 Km 227, 2300 Rafaela, Santa Fe Argentina; 7 Centro de Investigaciones en Abejas Sociales (CIAS)-Instituto de Investigación en Producción Sanidad y Ambiente (IIPROSAM CONICET-UNMdP), Facultad de Ciencias Exactas y Naturales, Centro Científico Tecnológico Mar del Plata-CONICET, Centro de Asociación Simple CIC PBA, Estación Costera J.J. Nágera, Ruta Provincial 11 Km 5395 Playa Chapadmalal (7603) Mar del Plata, Buenos Aires, Argentina; 8 Universidad Distrital Francisco José de Caldas, Facultad de Medio Ambiente y Recursos Naturales and Colectivo Abejas Vivas, Bogotá, Colombia; 9 Ministerio de la producción y ambiente Formosa (MPA), Facultad de Recursos Naturales, Universidad de Formosa (UNAF), Av Luís Gutnisky 3200, Formosa, Argentina; 10 Universidad SEK, Facultad de Ciencias de la Salud, Instituto de Investigación Interdisciplinar en Ciencias Biomédicas SEK, Santiago, Chile; 11 Área de Investigación y Desarrollo Tecnológico para la Agricultura Familiar Región Cuyo, INTA, San Juan entre Sarmiento y José Pedro Cortinez Oeste, San Martín, 5439 San Juan, Argentina; 12 Lab. de Microbiología y Salud de las Abejas, Departamento de Microbiología, Instituto de Investigaciones Biológicas Clemente Estable, Montevideo, Uruguay; 13 Instituto de Investigaciones para la Industria Química (INIQUI-CONICET), Universidad Nacional de Salta, Av. Bolivia 5150, Salta, Argentina; 14 Facultad de Ciencias Veterinarias-PROANVET Universidad Nacional del Centro de la Provincia de Buenos Aires UNCPBA, Tandil, Buenos Aires, Argentina; 15 Sección Apicultura, Instituto Nacional de Investigación Agropecuaria, Ruta 50, km 11, Colonia, Uruguay; 16 Programa Integrado de Patología Apícola, Centro de Investigaciones Apícolas Tropicales, Universidad Nacional, Heredia, Costa Rica; 17 Dep. de Ciências Animais, Universidade Federal Rural do Semi-Arido, Mossoró, RN Brazil; 18 Universidad de Guadalajara, Centro Universitario de la Costa Sur, Autlán, Jalisco México; 19 Departamento de Medicina y Zootecnia de Abejas, Conejos y Organismos Acuáticos, Facultad de Medicina Veterinaria y Zootecnia de la Universidad Nacional Autónoma de México, Ciudad Universitaria, Delegación Coyoacán, 04510 Mexico City, Mexico; 20 Universidade Federal de Sergipe, Campus do Sertão, Departamento de Educação em Ciências Agrárias e da Terra, e Instituto Nacional de Ciência e Tecnologia em Estudos Interdisciplinares e Transdisciplinares em Ecologia e Evolução (INCT-IN-TREE), Mossoró, Brazil; 21 Federación Colombiana de Apicultores y Criadores de Abejas, Bogota, Colombia; 22 Centro Integrado de Pesquisas Biotecnológicas, Campus São Gabriel, Universidade Federal do Pampa (UNIPAMPA), Rua Aluízio Barros Macedo, Br 290, km 423 Bairro Piraí, São Gabriel, RS 97300-000 Brazil; 23 Centro de Biodiversidad y Descubrimiento de Drogas, Instituto de Investigaciones Científicas y Servicios de Alta Tecnología (INDICASAT-AIP), Clayton, 0843-01103 Panamá; 24 Departamento de Zootecnia, Centro de Ciências Agrárias, Universidade Federal do Ceará, Fortaleza, CE 60356-000 Brazil; 25 Instituto de Ecología Regional (IER-CONICET), Tucumán, Argentina; 26 Centro Pyme Adeneu, Agencia de desarrollo económico del Neuquen, Neuquén, Argentina; 27 Department of Biology, University of Puerto Rico Rio Piedras Campus and Institute of Neurobiology, Medical Sciences Campus, San Juan, Puerto Rico; 28 Departamento de Biologia, Faculdade de Filosofia Ciências e Letras de Ribeirão Preto, Universidade de São Paulo, Ribeirão Prêto, SP Brazil; 29 Departamento de Producción Animal, Universidad de Buenos Aires, Facultad de Agronomía, Buenos Aires, Argentina; 30 INTA, Centro de Investigación en Recursos Naturales, Instituto de Recursos Biológicos, Buenos Aires, Argentina; 31 Associação de Proteção às Abelhas Bee or not to Be, Ribeirão Prêto, SP Brazil; 32 Universidad Nacional de Río Negro, Sede Andina, Escuela de Producción Agropecuaria y Tecnología Ambiental, El Bolsón, Argentina; 33 Estación Experimental Agropecuaria INTA Cuenca del Salado, Agencia de Extension Rural Chascomus, Buenos Aires, Argentina; 34 Director de Producción Apícola del Ministerio del Agro y de la Producción de la Provincia de Misiones. Centro de Investigación Apícola y Meliponícola del Instituto Superior del Profesorado en Ciencias Agrarias y Protección Ambiental (PROCAyPA), Misiones, Argentina; 35 Unidad Académica de Animales de Granja, Facultad de Veterinaria, Universidad de la República, Montevideo, Uruguay; 36 Instituto de Innovación para la Producción Agropecuaria y el Desarrollo Sostenible (IPADS) Balcarce (INTA-CONICET), RN 226 km 73.5,7620, Balcarce, Buenos Aires, Argentina; 37 Facultad de Ciencias Agrarias, Universidad Nacional de Mar del Plata (FCA-UNMdP), Ruta 226km 73.5, Balcarce, 7620 Buenos Aires, Argentina; 38 Laboratorio de Investigaciones en Abejas, Departamento de biología, Facultad de Ciencias, Universidad Nacional de Colombia, sede Bogotá, Colombia; 39 Grupo de Investigaciones para la Gestión y Conservación de Servicios Ecosistémicos, Corporación para la Gestión de Servicios Ecosistémicos, Polinización y Abejas-SEPyA, Bogotá D.C., Colombia; 40 Facultad de Zootecnia, Universidad Nacional del Centro del Perú, Av. Mariscal Castilla N° 3909, El Tambo, Huancayo, Perú; 41 Embrapa Recursos Genéticos e Biotecnologia, Parque Estação Biológica, Avenida W5 Norte (Final), Caixa Postal 02372, Brasília, DF 70770-917 Brazil; 42 Centro de Microbiología Básica y Aplicada (CEMIBA), Facultad de Ciencias Veterinarias, Universidad Nacional de La Plata (UNLP) y Consejo Nacional de Investigaciones Científicas y Técnicas, La Plata (CCT-CONICET, La Plata), La Plata, Buenos Aires, Argentina; 43 Centro de Investigaciones Apícolas, Havana, Cuba; 44 Laboratorio de Ecología Química, Facultad de Química, Universidad de la República, Montevideo, Uruguay; 45 Facultad de Ciencias, Iguá 4225, 11400 Montevideo, Uruguay; 46 Instituto de Genética E. A. Favret, Instituto Nacional de Tecnología Agropecuaria (INTA), Consejo Nacional de Investigaciones Científicas y Técnicas (CONICET), Hurlingham, Buenos Aires, Argentina; 47 Sección Apicultura, INIA La Estanzuela, Colonia, Uruguay; 48 Centro de Investigaciones en Abejas (CIABE), Centro Universitario del Sur, Universidad de Guadalajara, Enrique Arreola Silva 883, Cd., Guzman, JAL Mexico; 49 Facultad de Ciencias Veterinarias y Pecuarias, Beeing Company, Departamento Ciencias Universidad de Chile, Avda. Santa Rosa 11315, La Pintana, 882080 Santiago, Chile; 50 Instituto de Biologia, Universidade Federal da Bahia, Campus de Ondina, Rua Barão de Geremoabo s/n, Salvador, BA 40170-210 Brazil; 51 Departamento de Ciencias Agronómicas y Recursos Naturales, Facultad de Ciencias Agropecuarias y Forestales, Universidad de La Frontera, Temuco, Chile

Correction to: *Scientific Reports* 10.1038/s41598-024-59513-6, published online 02 May 2024

The PDF version of this Article contained errors in Equations 1 and 2, where the equations were incompletely displayed. The correct equations are displayed below:

1 $$\text{No}.\text{ colonies alive }= a+b-c$$

2 $$\text{No}.\text{colonies lost }= a+b-c-d$$

The original Article has been corrected.

