# Supplementary material for: First large-scale study reveals important losses of managed honey bee and stingless bee colonies in Latin America
Source: Sci Rep. 2024 May 2;14:10079. doi: 10.1038/s41598-024-59513-6 (PMC11066017; doi:10.1038/s41598-024-59513-6)
Supplement: Supplementary file 1 — Supplementary Information. [file 41598_2024_59513_MOESM1_ESM.docx]

**First large-scale study reveals important losses of managed honey bee and stingless bee colonies in Latin America**

Requier, F. *et al.*

*Scientific Reports*

**Supplementary Information**

| **Content** |  | **pj** |
| --- | --- | --- |
| **Figure S1** | Number of colonies for honey bees and stingless bees, per participants’ profiles and Latin American country. | **2** |
| **Section S1** | Questionnaire used for the two-year survey of managed honey bee and stingless bee colonies in Latin America. | **3** |
| **Table S1** | Summary of the Linear Model performed to evaluate the effects of op participants’ profile, country, bee type, year, and the interaction between participants’ profiles and country on the operation size. | **5** |
| **Table S2** | Summary of the Generalized Linear Model performed to evaluate the effects of large-scale monitoring initiative, year, and the interaction between large-scale monitoring initiative and year on winter colony loss of honey bees. | **6** |

**Figure S1.** Number of colonies (log-transformed) for (A) honey bees and (B) stingless bees, per participants’ profiles (professional in black, semi-professional in red and hobbyist in green) and Latin American country. Dots represent the predicted value per participant’s profile and country with thick lines indicating the 95% CI (based on Linear Model predictions). Countries are ordered by the decreasing log-transformed number of colonies. Horizontal dashed lines represent the average value of number of colonies (log-transformed) for each participant’s profile across Latin America.

**
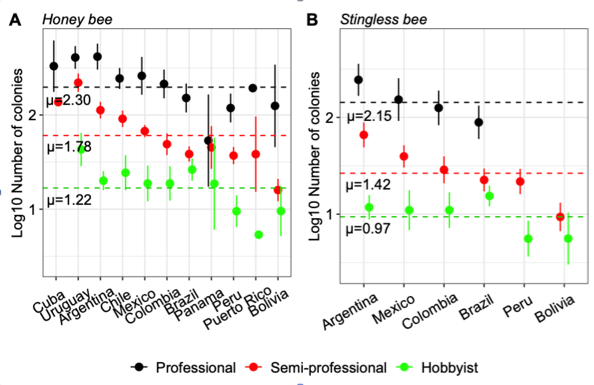
**

**Section S1.** Questionnaire used for the two-year survey (2016-2017 and 2017-2018) of managed honey bee and stingless bee colonies in Latin America. The questionnaire was offered in Spanish and Portuguese according to the country of residence of the participant, including: Argentina, Bolivia, Brazil, Chile, Colombia, Cuba, Mexico, Panama, Peru, Puerto Rico and Uruguay. The same question was used for beekeepers and meliponiculturists. The call for participation in the survey started in October until March each survey years (i.e. information for the 2016-2017 questionnaire was collected from October 2017 until March 2018, while information for 2017-2018 was collected from October 2018 until March 2019). Each participant (i.e. a beekeeper or meliponiculturist) was invited to answer specific questions about the (**A**) participant profile, (**B**) location of the main operation and (**C**) number of colonies.

**(A) Participant’s profile**: choosing their dedication to beekeeping between “Professional (beekeeping is the only source of income)”, “Semi-professional (beekeeping is important for family income, but has another complementary activity/profession)”, and “Breeder for family or personal consumption”. The last option corresponds to “Hobbyist”.

**(B) Location of the main operation**: country, state/province/region/department, nearest city/town, nearest zip code and coordinates (latitude, longitude).

**(C) Number of colonies**: for all 7 questions it was asked to provide information on the total number of alive colonies from all participant’s apiaries/meliponaries. We defined before the questions an “alive colony” as a colony having at least one queen, and brood and adult bee population covering at least four frames on both sides. The seven questions were the following:

(1) Number of colonies owned on October 1^st^, 2016 (“2017” for the second year).

(2) Number of colonies owned on April 1^st^, 2017 (“2018” for the second year).

(3) Number of alive colonies obtained by colony multiplication, purchase, or capture from October 2016 to April 2017 (“October 2017 to April 2018” for the second year).

(4) Number of colonies sold or given away from October 2016 to April 2017 (“October 2017 to April 2018” for the second year).

(5) Number of colonies owned on October 1^st^, 2017 (“2018” for the second year).

(6) Number of alive colonies obtained by colony multiplication, purchase, or capture from April 2017 to October 2017 (“April 2018 to October 2018” for the second year).

(7) Number of colonies sold or given away from April 2017 to October 2017 (“April 2018 to October 2018” for the second year). As mentioned before, we considered the operation size to be the annual number of colonies owned by the participant from the calculated number of alive and dead colonies annually (for further details see “Assessing bee colony loss”).

**Table S1**. Summary of the Linear Model performed to evaluate the effects of op participants’ profile, country, bee type, year, and the interaction between participants’ profiles and country on the operation size (log-transformed number of colonies). Participants’ profiles include professional, semi-professional and hobbyist. Bee types include honey bees and stingless bees. Bold lines indicate significant differences (*P*<0.05).

| Parameter | Sum Sq | Mean Sq | *F* value | *P* value |
| --- | --- | --- | --- | --- |
| **Participants’ profile** | **242.0** | **121.0** | **488.7** | **<0.001** |
| **Country** | **102.5** | **10.3** | **41.4** | **<0.001** |
| **Bee type** | **2.7** | **2.7** | **10.9** | **<0.001** |
| Year | 0.2 | 0.2 | 0.8 | 0.374 |
| **Operation type × Country** | **17.8** | **0.9** | **3.8** | **<0.001** |

**Table S2**. Summary of the Generalized Linear Model performed to evaluate the effects of large-scale monitoring initiative, year, and the interaction between large-scale monitoring initiative and year on winter colony loss of honey bees. Large-scale monitoring initiatives include Latin America (the present *SOLATINA* initiative), the United States (the *BIP* initiative) and Europe (the *COLOSS* initiative). Bold lines indicate significant differences (*P*<0.05).

| Parameter | Deviance | *F* value | *P* value |
| --- | --- | --- | --- |
| **Large-scale monitoring initiative** | **271907** | **190.7** | **<0.001** |
| Year | 105 | 0.1 | 0.701 |
| **Large-scale monitoring initiative × Year** | **5354** | **3.8** | **0.025** |
